# Returns handling in e-commerce: How to avoid demand negativity in supply chain contracts with returns?

**DOI:** 10.1007/s10660-023-09689-2

**Published:** 2023-03-20

**Authors:** Milena Bieniek

**Affiliations:** Economic Faculty, Institute of Management and Quality Sciences, Maria Curie-Sklodowska University, pl. Marii Curie-Sklodowskiej 5, Lublin, 20-031 Poland

**Keywords:** Supply chain management, Returns handling, Additive demand, E-commerce

## Abstract

E-commerce constitutes a system for the online purchase and sale of services and commodities. This theoretical article investigates the manufacturer handling strategy which occurs in online shopping, in a centralized or decentralized channel under the wholesale price contract. The retailer’s optimal order quantity, price, and the manufacturer’s wholesale price are derived assuming additive uncertainty in demand. The possibility of negative demand realizations, which may occur in adverse market circumstances, is verified in the investigated models. It was proved that the imposition of the non-negativity prerequisite on demand is vital to obtain complete results. The non-negativity constraint in this study incorporates consumer’s returns handling costs which is different than the previously used constraint. As an extension of the additive case, the model with iso-elastic demand is discussed. The theoretical analysis is enriched with numerical examples.

## Introduction

Recently, e-commerce has become an intrinsic part of the global retail structure. The retail market has undergone considerable changes motivated by the emergence of the Internet and the ever-present digitization of the modern life. Convenience, simplicity, information and time-efficiency of online shopping constitute properties appreciated by consumers [9]. The lack of geographical borders, optimized information and flow of goods, lower advertising and transaction costs are beneficial for businesses [51]. The number of online buyers has been steadily growing along with rapidly increasing global internet access and introduction of online solutions. In 2020, the year of the pandemic, over 2 billion people purchased merchandise and services online, and during the same year, e-retail sales exceeded $4.2 trillion worldwide and are foreseen to reach $6.54 trillion in 2023. Retail e-commerce traffic peaked at a record 22 billion monthly visits, with an immeasurably high demand for daily use products such as groceries, clothing and technical items. It is suspected that by 2040, 95% of retail purchases are likely to be made online and as a consequence, traditional retail may be hardly found (!) [10, 21]. The huge spike in total retail sales can come as a surprise taking into account the Covid-19 negative impact on in-store shopping. However, the growth in retail mainly originated from digital sales [4].

Offline sales are more sensitive to adverse market circumstances than e-commerce, but the wide swings in demand in online purchasing have also been observed recently [24]. Market fluctuations impacted by public trends, politics, terrorism, wars and currency devaluation will ultimately influence e-commerce [42]. E-business will also be affected by the variations of the global economy and consumer preferences [32]. Recently, massive-scale disruptions in the e-commerce industry caused by the Russo-Ukrainian war have been noted [8, 23].

The flood of returns constitutes a vital problem of e-commerce. It is definitely more serious than in the case of traditional sales. Research shows that online orders are returned by shoppers more than three times as frequently as when the same goods are purchased offline. The impact of returns handling in e-commerce is still underestimated [35]. The development of a suitable approach to returns has been a growing challenge for retailers and manufacturers [14]. It should be highlighted that one of the most common reason for returning online purchases in the U.S.A. is "Don’t like the items" which constitutes 37.7% of returns [27]. Consumers in numerous industries have a legal right to return their purchased products to original manufacturers or retailers for any reason [28]. False Failure Returns (FFR) are defined as returns of goods that have neither functional nor cosmetic defects [13]. It has been established that FFR constitute a major part of product returns [45, 50].

Motivated by the facts given above, the present article investigates the wholesale price contract with consumer returns caused by the mismatch when products fail to satisfy consumers’ expectations. We consider the reverse channel structure strategy in which the manufacturer handles and collects returns by itself and the exchange for a new variant of a product is allowed [39]. The manufacturer returns handling is especially suitable to e-commerce when the retailer is responsible for selling and the manufacturer provides after-sales service. For instance, if the consumer is not satisfied with mobile devices, e.g. Nokia and HTC mobiles purchased in the1stshop.com (n.d.) and YHD.com (n.d.), they can be returned and exchanged for a new product from the manufacturer’s local service stations. Similarly, if the consumer is unsatisfied with a Haier air conditioner purchased in Jingdong Mall the consumer return and exchange option is open in local after-sales service centers [25]. The wholesale price contract is used here, despite the fact that it cannot coordinate the supply chain, because of its simplicity and the fact that it is a commonplace practice in e.g. electronics [17], agriculture industry [26], and also used by large online retailers, e.g. Amazon [43].

Summarizing, we propose a general mathematical model to determine the optimal price and product quantity in purchasing under the return policy which according to the arguments may be of interest to electronic commerce. We extend the model of the wholesale price contract with returns introduced by Liu et al. [25]. In comparison with [25], the retail price is endogenous in our model and the stochastic demand is additive. This kind of demand is characterized by the fact that there exists an option that the actual demand is negative in adverse market conditions. The aim of the article is to modify the classical additive demand imposing on it the non-negativity constraint, and then obtain optimal solutions for centralized and decentralized channels. The main conclusion of this study is such that, in some cases, the optimal retail price is so high that the demand in the classical form may be negative. As a consequence, imposing the non-negativity assumption on demand is required to ensure the completeness of the considerations and avoid suboptimal solutions. As an extension, we give the respective results for the multiplicative demand. Illustrative numerical calculations were executed by means of the Mathematica software. It should also be noted that the solutions of the problem under the retailer returns handling strategy can be obtained in a similar way as those of the problem under the manufacturer handling strategy considered in this article.

## Literature

The present study refers to returns handling strategies and Operations Research problems associated with the non-negative demand. The subsequent paragraphs present the review of the recent literature concerning the above subjects.

### Returns handling strategy in the supply chain

Several articles assume that the consumer accepts the refund when the product mismatch occurs [12, 16]. In the model of Davis et al. [12], the profitability of money-back guarantee for a particular product is assessed by estimating salvage values of returned commodities, the probabilities of mismatching and transaction costs of returns. Hsiao and Chen [16] compare the profitability of two pervasively adopted return policies–money-back guarantee and hassle-free policies. The researchers who consider a consumer return policy in the case of the mismatch with permitted exchange are Ferguson et al. [13]; Ofek, Katona, and Sarvary [38]; Shulman, Coughlan, and Savaskan [30]. Ofek et al. [30] study how the physical store assistance level and the retailer’s pricing strategy change with the bonus in the online outlet in monopoly and duopoly settings. In some works, the manufacturer is responsible for handling inventory returns [25, 36, 38]. Shulman et al. [38] examine how the return penalty is affected by the choice of managing returns by the manufacturer or by the retailer. They argue that the manufacturer may earn more by accepting returns even if the retailer has a more efficient outlet for salvaging goods. J. Shi and Xiao [36] develop a game model of a vendor managed inventory supply chain to study the manufacturer’s consumer returns policy and the retailer’s store assistance service decision. They explore the effects of the supply chain decentralization and service subsidy rate on the returns policy. Liu et al. [25] explore returns handling strategies by means of the newsvendor framework. The authors focus on the reverse supply chain with demand uncertainty and provide an optimal order quantity in the model with the retailer or manufacturer returns handling strategy. They assume that the demand is price-free. Recently, the relationship between data-driven machine learning and cognitive decision-making algorithms as regards returns handling in e-commerce has been considered [15, 18, 19, 29]. It has been proved that becoming familiar with actual reasons for returns and with customer profitability through artificial intelligence gives opportunities to reduce online returns and their costs.

Our work extends the model of Liu et al. [25] for the manufacturer returns handling by adding price as a decision variable and considering price-dependent demand.

### Supply chain management under the non-negative additive demand

The problem of the theoretically negative realized demand in supply chain models is investigated in [5, 6, 20, 22]. Krishnan [20] emphasizes that the non-negativity assumption should be taken into account if the demand is in the additive form in order to provide the generality of the study. If the non-negativity constraint is not applied, the solution to the problem may become suboptimal. However, the non-negativity condition yields complications with the sufficiency of the first order conditions in a monopoly and the existence of equilibrium in an oligopoly. These threats have often been disregarded in existing research studies. Kyparisis and Koulamas [22] examine the price-setting newsvendor problem under the non-negative demand and prove that the issue always has an optimal solution, even in negative market circumstances. Bieniek [6] investigates a two-stage vendor managed consignment inventory contract with a similar restriction imposed on demand and demonstrates that the retailer’s expected profit does not have to be concave, but the problem always has at least one optimal solution. The newsvendor problem with barter exchange and the mean-variance newsvendor problem under demand non-negativity are considered in Bieniek [5] and Bieniek [7], respectively.

In this study, the additive stochastic demand is considered and therefore, in general, it may achieve negative values in its classical form. We offer the complete solution to the wholesale price contract with the manufacturer handling and additive demand which extends the considerations given in Liu et al. [25].

## General problem statement

We consider a supply chain with a manufacturer and a retailer with the availability of consumer returns. The manufacturer sells a new product to a consumer with the help of the retailer. The manufacturer accepts the consumer’s returns and, moreover, the consumer has the right to exchange the returned item for a new variant of the product. Model parameters and decision variables are listed in Table 1 and Basic assumptions in Table 2.

**Table 1:** Model parameters and notation

Decision variables	
*p*	Retail price per unit
*Q*	Order quantity
*z*	Inventory factor
*w*	Manufacturer’s wholesale price per unit
Notation	
*c*	Unit production cost
$h_m$	Manufacturer’s unit handling cost
$h_c$	Consumer’s average returns handling cost
$p+\alpha h_c$	Full price incurred by the consumer for a unit
$\alpha \in [0,1)$	Mismatch rate
$\varepsilon$	Random variable with expectation $\mu$, variance $Var (\varepsilon )<\infty$,
	cdf *F* and continuously differentiable pdf *f* with support [*A*, *B*]
*h*(*z*)	Failure rate, $h(z)=\frac{f(z)}{\bar{F}(z)}$, $\bar{F}(z)=1-F(z)$
*g*(*z*)	Generalized failure rate $g(z)=zh(z)$
$\bar{D}(p,\alpha )$	Expected demand
$X(p,\varepsilon )$	Stochastic demand
*a*, *b*	Demand parameters
$\mu (z)$	$\mu (z)=\int _z^B(z-\varepsilon )f(\varepsilon )d\varepsilon$, $z\in [A,B]$
$\Lambda (z)$	$\Lambda (z)=\int _{A}^z (z-\varepsilon )f(\varepsilon )d\varepsilon$, $z\in [A,B]$

**Table 2:** Basic assumptions

*F* is IFR (additive case)	
*F* is IGFR (multiplicative case)	
$\mu =0$ (WLOG), $A<0$ and $B>0$ (additive case)	
$\mu =1$ (WLOG), $0\le A<B$ (multiplicative case)	
Salvage value is equal = 0 (for simplicity)	
$A+a-bc-b\alpha (h_c+2h_m)>0$ (additive case)	
$c+\alpha h_m<p\le \frac{B+a-b\alpha h_c}{b}$ (additive case)	

We emphasize that the retail price is endogenous which is different than in [25] where price was assumed to be exogenous. The consumer’s taste is denoted by the mismatch rate. It is presumed that the returns come back to the manufacturer. Thereafter, the returned product can be resold as a new one through inspecting or repackaging. The consumer’s returns handling cost includes e.g. the customer’s reverse shipping fee, trip cost, time cost, and depends on the speed of response or convenience for consumer returns.

Let us define the price-dependent stochastic demand by $X(p,\varepsilon )$, where *p* is the price and $\varepsilon$ is the uncertainty parameter. The uncertainty is mainly included in the deterministic demand in the additive or multiplicative way. The additive stochastic demand is presented as

1 $$\begin{aligned} X(p,\varepsilon )=\bar{D}(p,\alpha )+\varepsilon \end{aligned}$$

and the multiplicative one as

2 $$\begin{aligned} X(p,\varepsilon )=\bar{D}(p,\alpha )\varepsilon , \end{aligned}$$

where $\bar{D}(p,\alpha )$ is the expected demand which is decreasing with the mismatch rate [30, 44].

First, the additive demand case is considered with

$$\begin{aligned} \bar{D}(p,\alpha )=a-b(p+\alpha h_c),\quad a,b>0. \end{aligned}$$

The realization of demand $X(p^{*},\varepsilon )=a-b(p^{*}+\alpha h_c)+\varepsilon$, where $p^{*}$ is the optimal price, can be negative if $p^{*}>p_{\max }$,

3 $$\begin{aligned} p_{\max }=\frac{A+a-b\alpha h_c}{b} \end{aligned}$$

and large negative $\varepsilon$ [20]. Thus, in this case, it is assumed that there is zero demand and we are going to use

4 $$\begin{aligned} X^+(p,\varepsilon )=(a-b(p+\alpha h_c)+\varepsilon )^+ \end{aligned}$$

[22] instead of the demand in the classical form (1). If $p>\frac{B+a-b\alpha h_c}{b}$ then $a-b(p+\alpha h_c)+\varepsilon <0$ for any realization of $\varepsilon$. Therefore, the considerations are confined to $p\le \frac{B+a-b\alpha h_c}{b}$.

The problem of demand negativity is different in nature than the bullwhip effect which describes how small fluctuations in demand at the retail level can cause progressively larger fluctuations in demand at the wholesale, distributor and manufacturer levels. This effect with regard to supply chain contracts have recently been studied in [1, 2, 48, 49].

As an extension to the additive case, optimization with the multiplicative demand in the form (2) with

5 $$\begin{aligned} \bar{D}(p,\alpha )=a(p+\alpha h_c)^{-b},\qquad a>0,\quad b>1, \end{aligned}$$

is conducted.

In the centralized channel, the manufacturer and the retailer co-operate and take a decision jointly. The central decision-maker decides retail price *p* and order quantity *Q* to maximize the total profit of the supply chain. Under the manufacturer handling, the expected profit is given by

6 $$\begin{aligned} \Pi _c(p,Q)=(p-\alpha h_m)\textrm{E}\min \{Q,X(p,\varepsilon )\}-cQ. \end{aligned}$$

In the decentralized supply chain, optimal decisions are made by the manufacturer and retailer independently maximizing their expected profits. The sequence of events is as follows. First, the manufacturer establishes the wholesale price and then, the retailer sets the retail price and order quantity.

We provide basic formulations of the expected profit functions of parties using the newsvendor framework. The manufacturer’s expected profit can be written as

$$\begin{aligned} \Pi _m(w\mid p,Q)=(w-c)Q-\alpha h_m\textrm{E}\min \{Q,X(p,\varepsilon )\}, \end{aligned}$$

and the retailer’s expected profit is given by

$$\begin{aligned} \Pi _r(p,Q\mid w)=p\textrm{E}\min \{Q,X(p,\varepsilon )\}-w Q. \end{aligned}$$

## Supply chains under the additive demand

In this section, we thoroughly analyze the existence of the negative additive actual demand in the studied centralized and decentralized supply chains. In the models $X^+(p,\varepsilon )$ given by (4) is applied instead of $X(p,\varepsilon )$.

### Centralized supply chain under the additive demand

We investigate the maximization problem

7 $$\begin{aligned} \max _{\begin{array}{c} c+\alpha h_m\le p\le \frac{B+a-b\alpha h_c}{b},\\ A\le z\le B \end{array}}\Pi _c(p,z)= (p-\alpha h_m)\textrm{E}\min \{Q,X^+(p,\varepsilon )\}-cQ \end{aligned}$$

in two cases: of always non-negative realizations of the additive demand and of possibly negative ones. Defining an inventory factor by $z=Q-\bar{D}(p,\alpha )$, where $z\in [A,B]$ [31], we obtain the expected profit equal to

$$\begin{aligned} \begin{aligned} \Pi _c(p,z)&=(p-\alpha h_m)\textrm{E}\min \{z+a-b(p+\alpha h_c),(a-b(p+\alpha h_c)+\varepsilon )^+\}\\&-c(z+a-b(p+\alpha h_c)). \end{aligned} \end{aligned}$$

#### Non-negative additive demand realizations in the centralized channel

In this case, we have $(a-b(p+\alpha h_c)+\varepsilon )^+=a-b(p+\alpha h_c)+\varepsilon$ for $p\le p_{\max }$ and any actual value of $\varepsilon \in [A,B]$. Hence, since $\textrm{E}\min \{z,\varepsilon \}=\mu (z)$, the expected profit is given by

$$\begin{aligned} \Pi _c(p,z)=(p-\alpha h_m)(\mu (z)+a-b(p+\alpha h_c)) -c(z+a-b(p+\alpha h_c)). \end{aligned}$$

We optimize

8 $$\begin{aligned} \max _{\begin{array}{c} c+\alpha h_m\le p\le p_{\max },\\ A\le z\le B \end{array}}\Pi _c(p,z) \end{aligned}$$

using the backward induction method [47] by first finding the optimal $p_c(z)$ for any given *z*, and then optimizing $\Pi _c(p_c(z),z)$ over *z* to discover $z_c$. We gain

$$\begin{aligned}{} & {} \frac{d\Pi _c(p,z) }{dp}=-2bp+\mu (z)+a+bc+b\alpha (h_m-h_c),\\{} & {} \quad \frac{d\Pi _c(p,z) }{dp}\mid _{p=c+\alpha h_m}>A+a-bc-b\alpha (h_c+h_m)>0, \end{aligned}$$

by Basic assumptions, and $\frac{d^2\Pi _c(p,z)}{dp^2}=-2b<0$. Then, from $\frac{d\Pi _c(p,z) }{dp}=0$ we see that the unique maximum is equal to

9 $$\begin{aligned} p_c(z)=\frac{\mu (z)+a+bc+b\alpha (h_m-h_c)}{2b}. \end{aligned}$$

It is important to establish whether $p_c(z)$ belongs to the interval $[c+\alpha h_m,p_{\max }]$. We relegate the proofs of the lemmas and theorems to the Appendix.

##### Lemma 1

Under Basic assumptions

1. $$p_c(z)\ge c+\alpha h_m$$;
2. $p_c(z)$ is an increasing and concave function of $z\in [A,B]$.

In view of the lemma, we cannot guarantee that $p_c(z)\le p_{\max }$. In this section we concentrate on prices not larger than $p_{\max }$ and because of that we define the hedged optimal price by

$$\begin{aligned} \beta _c(z)={\left\{ \begin{array}{ll} p_c(z) \text { for } z\le z_0,\\ p_{\max }\text { for } z> z_0 \end{array}\right. } \end{aligned}$$

[33], with $z_0\in [A,B]$ such that

$$\begin{aligned} \mu (z_0)=2A+a-bc-b\alpha (h_m+h_c) \end{aligned}$$

if $2A+a-bc-b\alpha (h_m+h_c)<0$ and $z_0=B$, otherwise. In order to optimize $\Pi _c(p_c(z),z)$, at the beginning, we redefine the objective function to

10 $$\begin{aligned} \Pi _c(z)=\Pi _c(\beta _c(z),z)={\left\{ \begin{array}{ll} \Pi _{c1}(z)=\Pi _c(p_c(z),z)\text { for } z\le z_0,\\ \Pi _{c2}(z)=\Pi _c\left( p_{\max },z\right) \text { for } z> z_0, \end{array}\right. } \end{aligned}$$

where $\Pi _{c2}(z)=(p_{\max }-\alpha h_m)(\mu (z)-A)-c(z-A)$. It should be stressed that $\Pi _c(z)$ is a continuous piecewise nonlinear function which by Lemma 1 consists of one or two pieces. Then, the first derivative of the objective function is

$$\begin{aligned} \Pi ^{'}_c(z)={\left\{ \begin{array}{ll} \Pi _{c1}^{'}(z)=(p_c(z)-\alpha h_m)\bar{F}(z)-c\text { for } z\le z_0,\\ \Pi _{c2}^{'}(z)=(p_{\max }-\alpha h_m)\bar{F}(z)-c\text { for } z> z_0, \end{array}\right. } \end{aligned}$$

which is smooth by taking the left and right derivatives at $z=z_0$ (at the point for which $p_c(z_0)=p_{\max }$). Then, the second derivative is equal to

$$\begin{aligned} \Pi ^{''}_c(z)={\left\{ \begin{array}{ll} \Pi _{c1}^{''}(z)=p_c^{'}(z)\bar{F}(z)-(p_c(z)-\alpha h_m)f(z)\text { for } z\le z_0,\\ \Pi _{c2}^{''}(z)=-(p_{\max }-\alpha h_m)f(z)\text { for } z> z_0. \end{array}\right. } \end{aligned}$$

We get the following useful lemma.

##### Lemma 2

Under Basic assumptions, function $\Pi _{c1}$ is first increasing and then concave on $[z_{1},B]$ for some $z_{1}\in [A,B]$. Moreover, $\Pi _{c2}$ is increasing-decreasing and concave on [*A*, *B*].

Consequently, we get the following theorem.

##### Theorem 1

Under Basic assumptions, the problem defined by (8) has a unique optimal solution

1. $(p_c(z_{c1}),z_{c1})$, where $p_c(z)$ is defined by (9) and $z_{c1}$ solves $(p_c(z)-\alpha h_m)\bar{F}(z)-c=0$ if $\bar{F}(z_0)<\frac{c}{p_{\max }-\alpha h_m}$;
2. $(p_{\max }, z_{c2})$, where $p_{\max }$ is defined by (3) and $z_{c2}$ solves $(p_{\max }-\alpha h_m)\bar{F}(z)-c=0$ if $\bar{F}(z_0)>\frac{c}{p_{\max }-\alpha h_m}$.

#### Negative additive demand realizations in the centralized channel

In this case, $p>p_{\max }$ and it is possible that the realized value of demand defined by (1) is negative. Therefore, now

$$\begin{aligned} \hat{\Pi }_c(p,z)=(p-\alpha h_m)(\mu (z)-\mu (b(p+\alpha h_c)-a)) -c(z+a-b(p+\alpha h_c)), \end{aligned}$$

and we solve the optimization problem

11 $$\begin{aligned} \max _{\begin{array}{c} p_{\max }\le p\le \frac{B+a-b\alpha h_c}{b},\\ b(p+\alpha h_c)-a\le z\le B \end{array}}\hat{\Pi }_c(p,z). \end{aligned}$$

In general, the derivations are mathematically complicated because of the form of $\mu (z)$. However, we can write it in a closed form for certain particular distributions, i.e. uniform, and gamma with specific parameters. Nevertheless, the issue still creates computational problems even for precise $\mu (z)$. However, we can establish the existence of a solution.

##### Theorem 2

The problem defined by (11) has an optimal solution which can be unique if $\hat{\Pi }_c(p,z)$ is concave with respect to *p* on the set $[p_{\max },\frac{B+a-b\alpha h_c}{b}]$.

#### Overall solution to the centralized channel under the additive demand

We conclude that the overall solution to the problem (7) is determined by

$$\begin{aligned} \max \left\{ \max _{\begin{array}{c} c+\alpha h_m\le p\le p_{\max },\\ A\le z\le B \end{array}}\Pi _c(p,z),\max _{\begin{array}{c} p_{\max }\le p\le \frac{B+a-b\alpha h_c}{b},\\ b(p+\alpha h_c)-a\le z\le B \end{array}}\hat{\Pi }_c(p,z)\right\} . \end{aligned}$$

### Decentralized supply chain under the additive demand

Now, we investigate the optimization problem for the decentralized channel in the two previously examined cases: if the actual additive demand is non-negative and if it could be negative.

#### Non-negative additive demand realizations in the decentralized channel

For $p\le p_{\max }$ we have the expected profits given by

$$\begin{aligned} \Pi _m(w\mid p,z)=(w-c)(a-b(p+\alpha h_c)+z)-\alpha h_m(a-b(p+\alpha h_c)+\mu (z)), \end{aligned}$$

and

$$\begin{aligned} \Pi _r(p,z\mid w)=p(a-b(p+\alpha h_c)+\mu (z))-w(a-b(p+\alpha h_c)+z). \end{aligned}$$

The backward induction method proposed by Zabel [47] will be used. First, we solve the retailer maximization problem

12 $$\begin{aligned} \max _{\begin{array}{c} c+\alpha h_m\le p\le p_{\max },\\ A\le z\le B \end{array}}\Pi _r(p,z\mid w) \end{aligned}$$

for a given wholesale price *w*. Namely, we determine the optimal $p_d$ for any given *z*, and then optimize $\Pi _r(p_d(z),z\mid w)$ over *z* to find $z_d$. We get

$$\begin{aligned} \frac{d\Pi _r(p,z\mid w) }{dp}=-2bp+a+bw-b\alpha h_c+\mu (z), \end{aligned}$$

$\frac{d^2\Pi _r(p,z\mid w)}{dp^2}<0$, $\frac{d\Pi _r(p,z\mid w)}{dp}\mid _{c+\alpha h_m}>A+a-bc-b\alpha (2h_m+h_c)>0$ by Basic assumptions which means that the function $\Pi _r$ is concave and increasing at the beginning with respect to *p*. The first order condition $\frac{d\Pi _r(p,z\mid w) }{dp}=0$ gives

13 $$\begin{aligned} p_d(z)=\frac{\mu (z)+a+bw-b\alpha h_c}{2b}. \end{aligned}$$

It is important to establish if $p_d(z)$ is hedged in the interval $[c+\alpha h_m,p_{\max }]$.

##### Lemma 3

Under Basic assumptions for a given *w*

1. $$p_d(z)\ge c+\alpha h_m$$;
2. $p_d(z)$ is an increasing and concave function of $z\in [A,B]$.

In view of the lemma we cannot guarantee that $p_d(z)\le p_{\max }$, thus, we define the hedged optimal price by

$$\begin{aligned} \beta _d(z)={\left\{ \begin{array}{ll} p_d(z) \text { for } z\le z_{0}^d(w),\\ p_{\max }\text { for } z> z_{0}^d(w), \end{array}\right. } \end{aligned}$$

where $z_0^d(w)\in [A,B]$ is given by

$$\begin{aligned} \mu (z_0^d(w))=2A+a-b\alpha h_c-bw \end{aligned}$$

if $2A+a-b\alpha h_c-bw\le 0$ and $z_0^d(w)=B$, otherwise. We redefine the objective function to

$$\begin{aligned} \Pi _r(z)=\Pi ^{}_r(\beta _d(z),z\mid w)={\left\{ \begin{array}{ll} \Pi _{r1}(z)=\Pi _r(p_d(z),z)\text { for } z\le z_0^d(w),\\ \Pi _{r2}(z)\text { for } z> z_0^d(w), \end{array}\right. } \end{aligned}$$

where

$$\begin{aligned} \Pi _{r2}(z)=p_{\max }(\mu (z)-A)-w(z-A). \end{aligned}$$

Consequently, $\Pi _r(z)$ is a continuous piecewise nonlinear function and its first derivative is presented as

$$\begin{aligned} \Pi _r'(z)={\left\{ \begin{array}{ll} \Pi _{r1}'(z)=p_d^{}(z)\bar{F}(z)-w \text { for } z\le z_0^d(w),\\ \Pi _{r2}'(z)=p_{\max }\bar{F}(z)-w \text { for } z> z_0^d(w), \end{array}\right. } \end{aligned}$$

which implies that it is also smooth. Moreover, by Lemma 3 we observe that $\beta _d(z)$ and $\Pi _r(z)$ consists of at most two pieces. The second derivative of $\Pi _r(z)$ is given by

$$\begin{aligned} \Pi ''_r(z)={\left\{ \begin{array}{ll} \Pi _{r1}''(z)=-\frac{f(z)}{2b}(\mu (z)+a+bw-bc\alpha h_c)+\frac{\bar{F}^2}{2b} \text { for } z\le z_0^d(w),\\ \Pi _{r2}''(z)=-p_{\max } f(z) \text { for } z> z_0^d(w). \end{array}\right. } \end{aligned}$$

We get the following lemma.

##### Lemma 4

Under Basic assumptions $\Pi _{r1}$ is first increasing and then concave for $z\in [z_{r1},B]$ for some $z_{r1}\in [A,B]$. $\Pi _{r2}$ is increasing-decreasing and concave on [*A*, *B*].

With the help of Lemma 4, we achieve the ensuing theorem.

##### Theorem 3

Under Basic assumptions, (12) has a unique optimal solution

1. $(p_{d}(z_{d1}),z_{d1})$, where $p_d(z)$ is given by (13) and $z_{d1}$ solves $p_{d}(z)\bar{F}(z)-w=0$ if $w\in B_1$;
2. $(p_{\max }, z_{d2})$ where $p_{\max }$ is defined by (3) and $z_{d2}$ solves $p_{\max }\bar{F}(z)-w=0$ if $w\in B_2$,

where $$B_1=\{w:\bar{F}(z_0^d(w))<\frac{w}{p_{\max }}\}$$ and $$B_2=\{w:\bar{F}(z_0^d(w))\ge \frac{w}{p_{\max }}\}$$.

Now, let us optimize $\max _{c\le w\le p}\Pi _m(w\mid p,z)$. The optimal solution exists according to the Extreme value theorem [34]. Our problem is to maximize

$$\begin{aligned} \Pi _m(w)={\left\{ \begin{array}{ll} \begin{aligned} &{}\Pi _{m1}(w\mid p_{d1},z_{d1})=(w-c)(a-b(p_{d}(z_{d1})+\alpha h_c)+z_{d1})\\ &{}-\alpha h_m(a-b(p_{d}(z_{d1})+\alpha h_c)+\mu (z_{d1})) \text { for } w\in B_1, \end{aligned}\\ \Pi _{m2}(w\mid p_{\max },z_{d2})=(w-c)(z_{d2}-A)-\alpha h_m(\mu (z_{d2})-A) \text { for } w\in B_2. \end{array}\right. } \end{aligned}$$

The first derivative of $\Pi _m(w)$ is equal to

$$\begin{aligned} \Pi _{m}'(w)={\left\{ \begin{array}{ll} \begin{aligned} &{}\Pi _{m1}'(w\mid p_{d}(z_{d1}),z_{d1})=a-bp_{d}(z_{d1}) -b\alpha h_c+z_{d1}\\ &{}+\frac{b}{2}(\alpha h_m-w+c) +\frac{(w-c)(2-\bar{F}(z_{d1}))-\alpha h_m\bar{F}(z_{d1})}{2}\frac{dz_{d1}}{dw}\\ &{}\text {for}\quad w\in B_1, \end{aligned}\\ \begin{aligned} &{}\Pi '_{m2}(w\mid p_{\max },z_{d2})=z_{d2}-A +\frac{c}{wh(z_{d2})} -\frac{p_{\max }-\alpha h_m}{p_{\max }h(z_{d2})}\\ &{}\text { for }\quad w\in B_2, \end{aligned} \end{array}\right. } \end{aligned}$$

where

$$\begin{aligned} \frac{dz_{d1}}{dw}=b\frac{2-\bar{F}(z_{d1})}{\bar{F}^2(z_{d1})-2bh(z_{d1})w}. \end{aligned}$$

We analytically specify feasible solutions which satisfy the first order condition $\Pi _{m}'(w)=0$. Then, an optimal solution is found numerically due to the mathematical complexity of the problem, namely $\Pi _{m1}$ and $\Pi _{m2}$ are optimized over $B_1$ and $B_2$ to choose optimal $w_{d1}\in B_1$ and $w_{d2} \in B_2$ if they exist. Finally, the optimal wholesale price is chosen, $w=w_{d1}$ if $\Pi _{m1}(w_{d1})> \Pi _{m2}(w_{d2})$ and $w=w_{d2}$, otherwise.

#### Negative additive demand realizations in the decentralized channel

In this case, $p>p_{\max }$ and it is possible that the realized value of (1) is negative. Consequently,

$$\begin{aligned} \hat{\Pi }_m(w\mid p,z)=(w-c)(z-b(p+\alpha h_c)+a)-\alpha h_m(\mu (z)-\mu (b(p+\alpha h_c)-a)) \end{aligned}$$

and

$$\begin{aligned} \hat{\Pi }_r(p,z\mid w)=p(\mu (z)-\mu (b(p+\alpha h_c)-a))-w(z-b(p+\alpha h_c)+a), \end{aligned}$$

and we solve the maximization problem

14 $$\begin{aligned} \begin{aligned} \max _{\begin{array}{c} p_{\max }\le p\le \frac{B+a-b\alpha h_c}{b},\\ b(p+\alpha h_c)-a\le z\le B \end{array}}\hat{\Pi }_r(p,z\mid w),\quad \max _{c\le w\le p}\hat{\Pi }_m(w\mid p,z). \end{aligned} \end{aligned}$$

In the decentralized channel, the derivations are also mathematically complicated because of the form of $\mu (z)$. However, we can solve the problem numerically.

##### Theorem 4

Under Basic assumptions, the maximization problem defined by (14) has a possibly non-unique optimal solution.

The optimal solution can be unique if the quasi-concavity of $\Pi _r$ and $\Pi _m$ can be shown and we have the ability to express $\mu (z)$ in an exact form.

#### Overall solution to the decentralized channel under the additive demand

We infer that the decentralized channel problem can be expressed as

$$\begin{aligned} \begin{aligned}&\max \left\{ \max _{w}\Pi _m(w\mid c+\alpha h_m\le p\le p_{\max },A\le z\le B),\right. \\&\quad \left. \max _{w}\hat{\Pi }_m(w\mid p_{\max }\le p\le \frac{B+a-b\alpha h_c}{b},b(p+\alpha h_c)-a\le z\le B)\right\} , \end{aligned} \end{aligned}$$

where *p* and *z* are the solutions to problems (12) and (14), respectively.

### Numerical examples in the additive case

This section outlines the numerical evidence which illustrates and verifies the preceding theoretical analysis. It is assumed that the demand function is given by $D(p,\varepsilon )=a-bp+\varepsilon$ where $\varepsilon \sim U[-A,A]$ which implies that $\mu (z)=\frac{(z+A)^2}{4A}$. The optimal solutions are printed in bold.

First, let us consider the centralized channel with the manufacturer handling strategy. Two sets of model parameters are used and they are specified in the tables. The parameters satisfy all of the Basic assumptions. The outcomes for the centralized channel are presented in Table 3. For the set of parameters $(a,b,c,A,\alpha ,h_c,h_m)=(35,1,1,-20,0.1,7,3)$, we infer that the problem given by (7) has a unique optimal solution which corresponds to the sub-problem with the possibly negative additive actual demand. It demonstrates that if we do not impose the non-negativity assumption, the solution will be suboptimal. For the next considered set of parameters, a unique optimal solution corresponds to the sub-problem with always positive additive actual demand.

**Table 3:** Solutions in the centralized supply chain with the additive demand, $\varepsilon \sim U[A,-A]$

$(a,b,c,A,\alpha ,h_c,h_m)$	$c+\alpha h_m\le p\le p_{\max }$	$p_{\max }\le p\le \frac{-A+a-b\alpha h_c}{b}$
$(35,1,1,-20,0.1,7,3)$	$p_c=14.7$	$\hat{p}_c=\mathbf {19.4125}$
	$z_c=17.1429$	$\hat{z}_c=\mathbf {17.8624}$
	$\Pi _c(z_c)=241.429$	$\hat{\Pi }_c(\hat{z}_c)=\mathbf {257.043}$
$(31,1,19,-10,0.1,7,3)$	$p_c=\mathbf {20.6772}$	$\hat{p}_c=20.7$
	$z_c=\mathbf {-9.0217}$	$\hat{z}_c=-9.00002$
	$\Pi _c(z_c)=\mathbf {0.500285}$	$\hat{\Pi }_c(\hat{z}_c)=0.5$

Next, let us consider the decentralized channel with the manufacturer handling strategy and the similar sets of parameters as in the centralized channel. The results are specified in Table 4. The conclusions are similar to those described for the centralized channel. Namely, for the first examined set of parameters the solution can be suboptimal if the non-negativity assumption is ensured. The optimal price is in the region of high prices which implies that the additive actual demand in the classical form may be negative. The second set of parameters creates the solutions in the region of small prices. All of these imply that the non-negativity prerequisite is essential to obtain generalized and correct solutions especially in the case of unfavorable economic conditions.

**Table 4:** Solutions in the decentralized supply chain with the additive demand, $\varepsilon \sim U[A,-A]$

$(a,b,c,A,\alpha ,h_c,h_m)$	$c+\alpha h_m\le p\le p_{\max }$	$p_{\max }\le p\le \frac{-A+a-b\alpha h_c}{b}$
$(35,1,1,-20,0.1,7,3)$	$p_d=14.7$	$\hat{p}_d=\mathbf {23.8897}$
	$z_d=-1.88153$	$\hat{z}_d=\mathbf {3.0372}$
	$w_d=8.04146$	$\hat{w}_d=\mathbf {10.1348}$
	$\Pi _m(w_d)=117.77$	$\hat{\Pi }_m(\hat{w}_d)=\mathbf {130.291}$
	$\Pi _r(p_d,z_d)=60.3213$	$\hat{\Pi }_r(\hat{p}_d,\hat{z}_d)=\mathbf {57.2077}$
$(31,1,19,-10,0.1,7,3)$	$p_d=\mathbf {20.6785}$	$\hat{p}_d=20.7$
	$z_d=\mathbf {-9.52929}$	$\hat{z}_d=-9.5086$
	$w_d=\mathbf {20.1918}$	$\hat{w}_d=20.1913$
	$\Pi _m(w_d)=\mathbf {0.245957}$	$\hat{\Pi }_m(\hat{w}_d)=0.2457$
	$\Pi _r(p_d,z_d)=\mathbf {0.12501}$	$\hat{\Pi }_r(\hat{p}_d,\hat{z}_d)=0.15322$

## Extension: model with the multiplicative demand

In this section, we examine supply chains with the multiplicative demand which complements the previous considerations.

### Centralized supply chain under the multiplicative demand

In the centralized channel, the expected profit is given by

15 $$\begin{aligned} \Pi _c(p,z)=\bar{D}(p,\alpha )((p-\alpha h_m)(z-\Lambda (z))-cz), \end{aligned}$$

after applying inventory factor $z=\frac{Q}{\bar{D}(p,\alpha )}$ [31] in (6). Here, $\bar{D}(p,\alpha )$ is defined by (5). The optimization problem

16 $$\begin{aligned} \max _{\begin{array}{c} c+\alpha h_m\le p,\\ A\le z\le B \end{array}}\Pi _c(p,z). \end{aligned}$$

is solved by first finding the optimal $p_c(z)$ for any given *z*, and then optimizing $\Pi _c(p_c(z),z)$ over *z* to determine $z_c$ according to the backward induction method [47].

#### Theorem 5

Under Basic assumptions, the problem defined by (16) has a unique optimal solution

17 $$\begin{aligned} p_c(z_c)=\frac{bcz_c}{(b-1)(z_c-\Lambda (z_c))}+\alpha \frac{bh_m+h_c}{b-1}, \end{aligned}$$

and $z_c$ given by $(p_c(z)-\alpha h_m)\bar{F}(z)-c=0$.

### Decentralized supply chain under the multiplicative demand

In the decentralized channel under the multiplicative demand, the expected profits are given by

18 $$\begin{aligned} \Pi _m(w\mid p,z)=\bar{D}(p,\alpha )((w-c)z-\alpha h_m(z-\Lambda (z))), \end{aligned}$$

and

19 $$\begin{aligned} \Pi _r(p,z\mid w)=\bar{D}(p,\alpha )(p(z-\Lambda (z))-wz). \end{aligned}$$

The backward induction method will be employed [47]. First, we solve the retailer maximization problem

20 $$\begin{aligned} \max _{\begin{array}{c} c+\alpha h_m\le p,\\ A\le z\le B \end{array}}\Pi _r(p,z\mid w) \end{aligned}$$

for a given wholesale price *w*. We find the optimal $p_d(z)$ for any given *z*, and optimize $\Pi _r(p_d(z),z)$ over *z* to decide $z_d$.

#### Theorem 6

For a given *w* under Basic assumptions, the problem defined by (20) has a unique optimal solution $(p_{d}(z_d),z_{d})$, where

21 $$\begin{aligned} p_{d}(z)=\frac{bwz}{(b-1)(z-\Lambda (z))}+\frac{\alpha h_c}{b-1} \end{aligned}$$

and $$z_{d}$$ solves

22 $$\begin{aligned} p_{d}(z)\bar{F}(z)-w=0. \end{aligned}$$

Next, we optimize $\Pi _m(w)$ defined by (18) over *w* provided that the optimal price and inventory factor are known. For that reason, our problem is to maximize

23 $$\begin{aligned} \max _{c\le w\le p}\Pi _m(w), \end{aligned}$$

where $\Pi _m(w)=\Pi _m(w\mid p_d,z_d)$ and $(p_d,z_d)$ is specified by (21) and (22), respectively. The optimal solution always exists according to the Extreme value theorem [34]. We obtain the following lemma

#### Lemma 5

Possible optimal wholesale price $w_d$ satisfies the first order condition

24 $$\begin{aligned} \begin{aligned}&\frac{z_d+(w-c-\alpha h_m\bar{F}(z_d))\frac{dz_d}{dw}}{(w-c)z_d-\alpha h_m(z_d-\Lambda (z_d))}\\&\quad -\frac{b^2(z_d(z_d-\Lambda (z_d))+w(z_d-\Lambda (z_d)-z_d\bar{F}(z_d))\frac{dz_d}{dw})}{(p_d(z_d)+\alpha h_c)(b-1)(z_d-\Lambda (z_d))^2}=0, \end{aligned} \end{aligned}$$

where

$$\begin{aligned} \frac{dz_d}{dw}=\frac{(z_d-\Lambda (z_d))\{(b-1)(z_d-\Lambda (z_d))-bz_d\bar{F}(z_d)\}}{w\{b\bar{F}(z_d)(z_d-\Lambda (z_d)-z_d\bar{F}(z_d))-(b-1)(z-\Lambda (z_d))^2h(z_d)\}}, \end{aligned}$$

and $(p_d,z_d)$ specified by (21) and (22).

Because of the mathematical complexity of (24), the unique solution to problem (23), if it exists, can be found numerically.

### Numerical examples in the multiplicative case

**Fig. 1:**
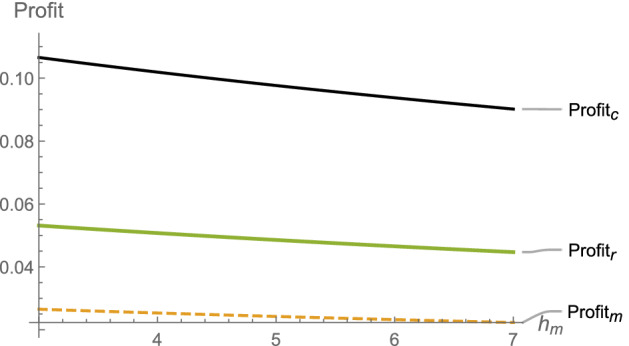
Dependence of profits on the manufacturer unit handling cost in the centralized ($Profit_c$) and decentralized ($Profit_r$ and $Profit_m$) channel

**Fig. 2:**
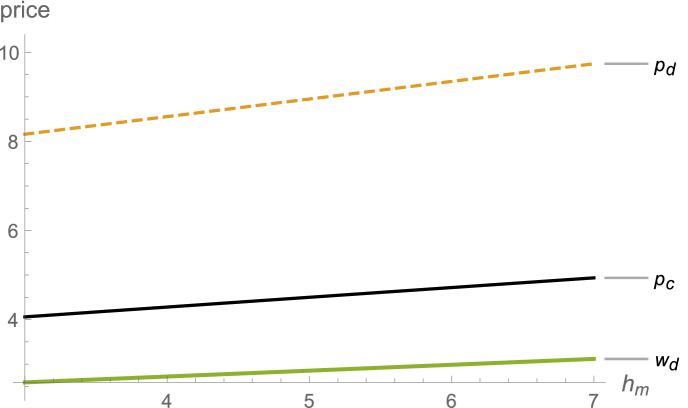
Dependence of prices on the manufacturer unit handling cost in the centralized ($p_c$) and decentralized channel ($p_d$ and $w_d$)

**Fig. 3:**
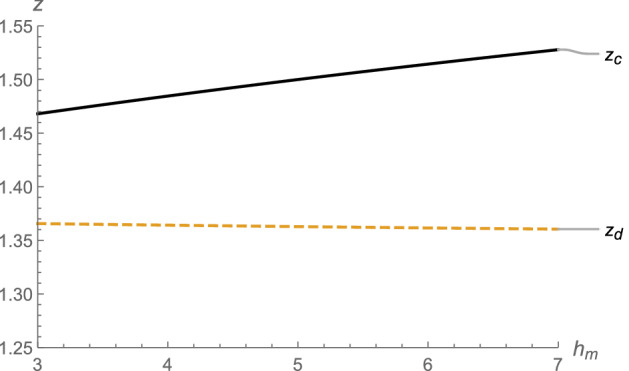
Dependence of inventory factors on the manufacturer unit handling cost in the centralized ($z_c$) and decentralized channel ($z_d$)

We illustrate and verify the preceding theoretical analysis for the multiplicative demand. It is assumed that the random part of demand follows the IGFR uniform distribution on [0, *B*]. Let us consider iso-elastic demand function $\bar{D}(p,\varepsilon )=ap^{-b}\varepsilon$. The values of other parameters are given in Table 5 along with numerical results for the centralized and decentralized channel. Obviously, the outcomes show that the wholesale price contract does not coordinate the supply chain. However, as we explain in the Introduction, it is still quite frequently used in practice. The sensitivity analysis with respect to the manufacturer’s handling cost is illustrated in figures. The expected profits in both centralized and decentralized channels decrease if the manufacturer’s returns handling cost increases (Fig. 1). In the centralized channel the expected profit decreases as the manufacturer’s handling cost increases despite the fact that, at the same time, the retail price and inventory factor increase (Figs. 2, 3). In the decentralized case, a higher manufacturer’s handling cost increases the retail and wholesale prices (Fig. 2) and, simultaneously decreases the inventory factor (Fig. 3) which implies the lower manufacturer and retailer profits.

**Table 5:** Solutions in the centralized and decentralized supply chain with the multiplicative demand

$(a,b,c,B,\alpha ,h_m,h_c)$	Centralized	Decentralized
(1, 2, 1, 2, 0.1, 7, 3)	$p_c=4.9361$	$p_d=9.7440$
	$z_c=1.5279$	$z_d=1.3604$
		$$w_d=3.1106$$
	$\Pi (z_c)=0.0902$	$\Pi _m(w_d)=0.0223$
		$$\Pi _r(p_d,z_d)=0.0447$$

## Discussion

Our work extends the model of Liu et al. [25] for the manufacturer returns handling by adding price as a endogenous variable. In Liu et al. [25], the retail price is given in advance and the consumer demand is stochastic and price-free. Compared to that work, in this article the demand function is not only stochastic but also linearly price-sensitive. Moreover, demand expression incorporates additive uncertainty which implies that theoretically demand may take negative values in a disadvantageous market situation. Under these assumptions, we offer the complete solution to the wholesale price contract under the manufacturer returns handling strategy proposed in Liu et al. [25]. The study shows that the non-negativity limitation is indispensable to avoid suboptimal solutions and to ensure the completeness of the discussion. These statements are in line with the results of Kyparisis and Koulamas [22], Bieniek [6], Bieniek [5] and Bieniek [7] where other supply chain models were studied. Kyparisis and Koulamas [22], Bieniek [5] and Bieniek [7] consider the newsvendor problem of various kinds and Bieniek [6] investigates vendor managed consignment inventory contract which is similar to this study.

## Conclusions

The large volume of online returns has become a massive problem for e-commerce, e.g. for some fashion products return rates run at up to 40%. Seeking models regarding the supply chain with returns will remain a matter of urgent concern. In this theoretical work, we introduce a mathematical model to determine the optimal price and quantity of products in purchasing with returns. We investigate the wholesale price contract in the centralized and decentralized channel under the manufacturer returns handling strategy which is introduced by Liu et al. [25]. In the centralized channel, the decision-maker sets the order quantity and price. In the decentralized channel the manufacturer and retailer play a Stackelberg game. First, the manufacturer offers a wholesale price and if the retailer accepts the offer, it determines the order quantity and price. The additive demand is addressed because it has a particular trait which is the opportunity that the actual demand records negative values. We present optimal solutions for the centralized and decentralized supply chain after imposing the non-negativity constraint on demand. The solutions to the problems can be suboptimal without this restriction, and for that reason, it is required in order to arrive at general findings. As an extension to the reflections on models with the additive demand, we study the cases with the multiplicative demand. The numerical examples illustrate the theoretical results well. The sensitivity analysis with respect to the manufacturer’s handling cost is done for the multiplicative case.

Our exploration may have several managerial implications. The theoretical model presented in this article is very general. Since e-commerce suffers from a very large volume of cumbersome returns and often uses a wholesale price contract, the model may attract e-commerce market participants. Under the additive demand, this paper helps online retailers determine the optimal pricing of products with FFR in the case of adverse market conditions, i.e. during a war or pandemic. Considering the multiplicative demand, this paper gives optimal quantities to retailers and manufacturers if FFR exist and if this kind of demand is applied e.g. high-fashion or new products’ demand [3], electricity demand [37], demand for air tickets [11].

There are also some limitations of the study. Optimization problems for centralized and decentralized channels with the non-negativity assumption are mathematically complicated and the analytical solutions cannot be specified. Due to this mathematical complexity, the design of effective algorithms is needed to explore the limits. However, this is outside the scope of this article and can be a subject of future studies. Moreover, one can consider the problem of demand non-negativity in other contracts between market players as a new direction of research. Finally, specific electronic commerce scenarios or organizations can be found in which the results of the article would be applicable.

## Appendix A

All proofs are based on the methods of calculus and the classical theory of maxima [34, 41].

### Proof of Lemma 1

By (17) and Basic assumptions we have

$$\begin{aligned} \begin{aligned} p_c(z)&\ge p_c(A)\ge \frac{A+a+bc+b\alpha h_m-b\alpha h_c}{2b}\\&=c+\alpha h_m+\frac{A+a-bc-b\alpha (h_m+ h_c)}{2b}\ge c+\alpha h_m, \end{aligned} \end{aligned}$$

which ends the proof of 1. The second formulation of the lemma follows from the fact that $\mu (z)$ is increasing. $\square$

### Proof of Lemma 2

$\Pi _{c1}$ is a continuous function with the first derivative $\Pi _{c1}'(A)=p_c(A)-\alpha h_m-c>0$ which proves that it is increasing at $z=A$. Moreover, $\Pi _{c1}''(z)<0$ if $h(z)>\frac{1}{A+a+bc-bc(h_m+h_c)}$, which by IFR property of distribution *F* is true for some $z>z_1$, $z_1\in [A,B]$. This means that additionally taking into account the Basic assumptions, we get that function $\Pi _{c1}$ is concave in interval $[z_1,B]$.

Furthermore, the derivatives $\Pi _{c2}'(z)(A)=p_{\max }-\alpha h_m-c>0$ and $\Pi _{c2}'(z)(B)=-c<0$ which means that the function is increasing at $z=A$ and decreasing at $z=B$. Finally, the second derivative $\Pi _{c2}''(z)<0$ for any $z\in [A,B]$, which implies that the function is concave. The proof is complete. $\square$

### Proof of Theorem 1

The statements of the theorem follow from Lemma 2 noting that $$\Pi _c(z)$$ is a smooth function and $$z_{c1}$$ and $$z_{c2}$$ are determined by the first order condition $$\Pi '_c(z)=0$$. Let us observe that $$\Pi _c(z)$$ is increasing in $$z_0$$ if $$\bar{F}(z_0)>\frac{c}{p_{\max }-\alpha h_m}$$. Therefore, in this case the maximum is attained in the interval $$[z_0,B]$$. Otherwise, the maximum is attained in the interval $$[A,z_0)$$. The proof is complete. $$\square$$

### Proof of Theorem 2

The assertion follows from the Extreme value theorem [34]. $\square$

### Proof of Lemma 3

By (13) and Basic assumptions we have

$$\begin{aligned} \begin{aligned} p_d(z)&\ge p_d(A)\\&\ge c+\alpha h_m+\frac{A+a-bc+b(w-c)-b\alpha (2h_m+ h_c)}{2b}\ge c+\alpha h_m, \end{aligned} \end{aligned}$$

which ends the proof of 1. The second formulation of the lemma follows from the fact that $\mu (z)$ is increasing. $\square$

### Proof of Lemma 4

The proof is similar to the proof of Lemma 2. Function $\Pi _{r1}$ is continuous and increasing in $z=A$ because $\Pi _{r1}'(A)=\frac{A+a-b\alpha h_c-bw}{2b}>0$ by the fact that $w<p_{\max }$. Moreover, $\Pi _{r1}'(B)=-w<0$, and by IFR property $\Pi _{r1}''<0$ for any $z>z_{r1}$, which implies the first statement of the lemma. Next, $\Pi _{r2}'(A)=p_{\max }-w>0$, $\Pi _{r2}'(B)=-w<0$ and $\Pi _{r2}''(z)=-p_{\max }f(z)<0$, which proves that $\Pi _{r2}(z)$ is first increasing then decreasing and concave. The proof is complete. $\square$

### Proof of Theorem 3

The conclusions of the theorem follow from Lemma 4 noting that $\Pi _r(z)$ is a smooth function and $z_{d1}$ and $z_{d2}$ are determined by the first order condition $\Pi '_r(z)=0$. Let us note that $\Pi _r(z)$ is increasing in $z_0^d$ if $w\in B_1$ and decreasing in $z_0^d$, otherwise, which points out the set to which the maximum belongs. $\square$

### Proof of Theorem 4

The assertion follows from the Extreme value theorem [34]. $\square$

### Proof of Theorem 5

From (15) we have

$$\begin{aligned} \frac{\textrm{d}\Pi _c(p,z)}{\textrm{d}p}=\frac{\bar{D}(p,\alpha )}{p+\alpha h_c}[-(b-1)(z-\Lambda (z))p+\alpha (z-\Lambda (z))(bh_m+h_c)+bcz], \end{aligned}$$

which by the first order condition $\frac{\textrm{d}\Pi _c(p,z)}{\textrm{d}p}=0$ gives the formula for optimal price $p_c(z)$ given by (17). Let us remark that $\frac{\bar{D}(p,\alpha )}{p+\alpha h_c}>0$. The solution $p_c(z)$ is unique since the gradient $-(b-1)(z-\Lambda (z))<0$ of the linear function of *p* in square brackets which means that it is decreasing. Therefore, $\frac{\textrm{d}\Pi _c(p,z)}{\textrm{d}p}>0$ for $p<p_c(z)$ and $\frac{\textrm{d}\Pi _c(p,z)}{\textrm{d}p}<0$ for $p>p_c(z)$. Next, substituting formula (17) into formula (15) we get $\Pi _c(p_c(z),z)=\Pi _c(z)$, which is a continuous and smooth function. Then, the optimal solution $z_c$ is determined by the first order condition $\Pi _c'(z)=\bar{D}(p,\alpha )((p-\alpha h_m)\bar{F}(z)-c)=0$. Furthermore, $\Pi _c'(z)$ can be written as

$$\begin{aligned} \Pi _c'(z)=\frac{c\bar{D}(p,\alpha )}{(b-1)(z-\Lambda (z))}H(z), \end{aligned}$$

where

$$\begin{aligned} H(z)=z-bzF(z)+\frac{\alpha }{c}(h_m+h_c)(z-\Lambda (z))\bar{F}+(b-1)\Lambda (z). \end{aligned}$$

Then

$$\begin{aligned} H'(z)=\bar{F}(z)[1-bzh(z)+\frac{\alpha }{c}(h_m+h_c)\bar{F}(z) -\frac{\alpha }{c}(h_m+h_c)h(z)(z-\Lambda (z))], \end{aligned}$$

and

$$\begin{aligned} H''(z)\mid _{H'(z)=0}=-\bar{F}(z)[bg'(z)+\frac{\alpha }{c}(h_m+h_c)f(z)+ \frac{\alpha }{c}(h_m+h_c)h'(z)(z-\Lambda (z))]<0 \end{aligned}$$

by Basic assumptions. This implies that *H*(*z*) is unimodal, increasing in *A* since $H(A)=A(\frac{\alpha }{c}(h_m+h_c)+1)>0$ and decreasing in *B* because $H(B)=-(b-1)<0$. Thus, we achieve that $z_c\in [A,B]$ is a unique maximum. The proof is complete. $\square$

### Proof of Theorem 6

Using formula (19) for a given $z\in [A,B]$ we have

$$\begin{aligned} \frac{\textrm{d}\Pi _r(p,z\mid w)}{\textrm{d}p}=\frac{\bar{D}(p,\alpha )}{p+\alpha h_c}[-(b-1)(z-\Lambda (z))p+\alpha h_c(z-\Lambda (z))+bcz+bwz], \end{aligned}$$

which by the first order condition $\frac{\textrm{d}\Pi _r(p,z\mid w)}{\textrm{d}p}=0$ gives the formula for optimal price $p_d(z)$ defined by (21). Note that $\frac{\bar{D}(p,alpha)}{p+\alpha h_c}>0$. The solution is unique since the gradient of a line $-(b-1)(z-\Lambda (z))<0$, and therefore, $\frac{\textrm{d}\Pi _r(p,z\mid w)}{\textrm{d}p}>0$ for $p<p_d(z)$, and $\frac{\textrm{d}\Pi _r(p,z\mid w)}{\textrm{d}p}<0$ for $p>p_d(z)$. Next, substituting expression (21) to formula (19) we get $\Pi _r(p_d(z),z\mid w)=\Pi _r(z\mid w)$, which is a continuous function of *z* with the first derivative equal to $\Pi _r'(z\mid w)=\bar{D}(p,\alpha )(p_d(z)\bar{F}(z)-w)$. The optimal $z_d(w)$ is given by the first order condition $\Pi _r'(z\mid w)=0$. Let us note that $\Pi _r'(z\mid w)$ can be written as $\Pi _r'(z\mid w)=\frac{w\bar{D}(p,\alpha )}{z-\Lambda (z)}H(z)$, where

$$\begin{aligned} H(z)=z-bzF(z)+(b-1)\Lambda (z)+\frac{\alpha }{w}h_c\bar{F}(z)(z-\Lambda (z)). \end{aligned}$$

Then $$\frac{w\bar{D}(p,\alpha )}{z-\Lambda (z)}>0$$, $$H'(z)=\bar{F}(z)[1-bzh(z)-\frac{\alpha }{w}h_ch(z)(z-\Lambda (z))+\frac{\alpha }{w} h_cF(z)]$$ and

$$\begin{aligned} H''(z)\mid _{H'(z)=0}=-\bar{F}(z)[bzh'(z) +\frac{\alpha }{w}h_ch'(z)(z-\Lambda (z)) +\frac{\alpha }{w}h_ch(z)\bar{F}(z)+\frac{\alpha }{w}h_cf(z)] \end{aligned}$$

is negative which means that for any $z\in [A,B]$, such that $H'(z)=0$, the function *H*(*z*) is concave. Moreover, $H(A)=A+\frac{\alpha }{w}h_cA>0$ implies that *H* increases in *A* and $H(B)=-(b-1)<0$ implies that *H* decreases in *B*. This means that *H*(*z*) is unimodal and $z_d(w)\in [A,B]$ is a unique maximum. The proof is complete. $\square$
